# Evaluation the efficacy of oral immunization of broiler chickens with a recombinant *Lactobacillus casei* vaccine vector expressing the Carboxy-terminal fragment of α-toxin from *Clostridium perfringens*

**DOI:** 10.1186/s12917-023-03566-8

**Published:** 2023-01-19

**Authors:** Mohammad Ali Shamshirgaran, Mehdi Golchin, Mahmoud Salehi, Reza Kheirandish

**Affiliations:** 1 Division of Microbiology, Department of Pathobiology, Faculty of Veterinary Medicine, Shahid Bahonar University of Kerman, Post Box: 76169-133, Kerman, 7616914111 Iran; 2 Division of Poultry Diseases, Department of Clinical Science, Faculty of Veterinary Medicine, Shahid Bahonar University of Kerman, Kerman, Iran; 3 Division of Pathology, Department of Pathobiology, Faculty of Veterinary Medicine, Shahid Bahonar University of Kerman, Kerman, Iran

**Keywords:** α-Toxin, *Clostridium perfringens*, *Lactobacillus casei*, Necrotic enteritis, Probiotic, Vector vaccine

## Abstract

**Background:**

*Clostridium perfringens* (*C. perfringens*) is a serious anaerobic enteric pathogen causing necrotic enteritis (NE) in broiler chickens. Following the ban on antibiotics as growth promoters in animal feedstuffs, there has been a remarkable rise in occurrence of NE which resulted in considering alternative approaches, particularly vaccination. The objective of this work was to evaluate the recombinant *Lactobacillus casei* (*L. casei*) expressing the C-terminal domain of α-toxin from *C. perfringens* as a potential probiotic-based vaccine candidate to immunize the broiler chickens against NE.

**Results:**

The broiler chickens immunized orally with recombinant vaccine strain were significantly protected against experimental NE challenge, and developed specific serum anti-α antibodies. Additionally, the immunized birds showed higher body weight gains compared with control groups during the challenge experiment.

**Conclusions:**

The current study showed that oral immunization of broiler chickens with a safe probiotic-based vector vaccine expressing α-toxin from *C. perfringens* could provide protective immunity against NE in birds.

**Supplementary Information:**

The online version contains supplementary material available at 10.1186/s12917-023-03566-8.

## Background

Necrotic enteritis (NE), a devastating disease of chickens and turkeys was first described by Parrish in 1961 in England [1]. Approximately $6 billion in economic losses are caused by this disease every year worldwide, affecting all poultry-producing countries [2, 3]. NE can be divided into two forms: clinical and subclinical [1]. The clinical or acute form is related to a short period of involvement with signs of depression, diarrhea, sternal recumbency, and severe necrosis in the mucosa of the small intestine. Subclinical or chronic NE is related to a reduction in feed intake and body weight gain of birds. This form is pathologically attributed to extensive damage in the mucosa of the small intestine of the NE-affected birds [4–6]. NE typically affects birds in excellent body condition [4], with a mortality rate as high as 50% [7]. The birds are most susceptible to NE at 2-to-6 weeks of age, but in commercial layers, it has been reported in birds over 3 months old [6].

*Clostridium perfringens* (*C. perfringens*), an anaerobic gram-positive and spore-forming bacterium, is broadly distributed in the environment, being present in soil, in decaying organic matter, and is a member of the intestinal normal microbiota of many humans and animals [8]. This bacterium had been divided into seven types (A to G) based on major toxins of α, β, ε, ι, CPE and necrotic enteritis toxin β-like (NetB) [9, 10]. *C. perfringens* type G, formerly described under type A, is the principal causative agent of NE, producing α-toxin and NetB toxin [10].

*C. perfringens*-caused infections such as NE were routinely prevented by using antimicrobial agents. However, due to many concerns have raised about subsequent antibiotic resistance as an adverse effect of widely used antibiotics for public health, the European union entirely banned the usage of the antibiotics as growth promoters in 2006, which consequently resulted in a significant increase in the occurrence of the several diseases in animals especially NE in broiler chickens [11, 12].

Following the withdrawal of the antibiotics, several studies focused on alternative control methods such as vaccination of the birds to induce protective immunity against experimental induction of NE by using different immunogenic antigens of *C. perfringens*. Some studies formerly showed that different immunogenic antigens of *C. perfringens* could elicit protective immune responses in broiler chickens [13, 14]. The α-toxin is one of the most important toxins produced by NE-caused *C. perfringens*, and the capability of this toxin in eliciting protective immune responses against NE in birds challenged with virulent *C. perfringens* was proved in several previous studies. The Carboxy-terminal (C-terminal) fragment of α-toxin from *C. perfringens* was previously recognized as an immunogenic antigen by vaccination of the animal models, evaluation of the immune responses, and assessment of the protection against the experimental challenge [15–19]. It is also indicated that the C-terminal fragment of α-toxin could elicit humoral and cell-mediated immune responses and provide significant protection in several vaccine studies against the *C. perfringens* toxin challenge in mice [15–17] and the experimental challenge of NE in broiler chickens [18, 19].

Recently, the usage of live oral vaccines has dramatically increased due to convenient route of administration, less expense, induction of protective mucosal cell-mediated immunity as well as systemic immunity against immunogenic antigens of *C. perfringens*, and the ability to express more than one foreign immunogen in a single vector [20, 21]. Several studies formerly showed that vaccination of birds with live oral vaccines expressing the variety of *C. perfringens* antigens could induce specific antitoxin immune responses and also lower the severity of NE-related intestinal lesions, as well as the number of NE-affected birds followed by the experimental challenge [13, 14, 18, 22].

Moreover, many investigators have already showed that supplementation of probiotics or direct-fed microbial (DFM), as captivating live microbial feed additives, in chicken’s diet could reduce the severity of NE lesions in the small intestine of birds and likewise, regulate the intestinal mucosal immunity as well as prevention of pathogen colonization [21, 23, 24]. Additionally, probiotics could serve as promising vaccine vectors to deliver antigens due to some advantages such as survival for a long time in the body, lack of toxicity, and convenient cultivation [25]. Among all, *Lactobacillus* species as the safe probiotic bacteria [26], have already been used as the safe vaccine vector to deliver the different antigens and also served as a promising vaccine candidate against some experimentally induced diseases in birds [27–30]. In the current study, we aimed to investigate, for the first time, the effectiveness of the recombinant *Lactobacillus casei* (*L. casei*) expressing the C-terminal domain of the α-toxin from *C. perfringens*, as a safe recombinant probiotic-based vaccine strain for oral immunization the broiler chickens against experimentally-induced NE.

## Results

### Protection against NE

Protection against the experimental challenge with virulent *C. perfringens* in birds was assessed based on gross intestinal lesions observed in necropsy, and also the changes in body weight gains during the experiment. Birds orally vaccinated with recombinant *L. casei* expressing C-terminal domain of the α-toxin (LC-α strain) were significantly protected against the experimental *C. perfringens* infection compared with birds received *L. casei* carrying the empty vector (LCP strain) and non-vaccinated (NV-challenged) birds (*P* < 0.05) (Table 1). The lesions observed in immunized birds with recombinant LC-α vector vaccine were reduced in severity and frequency, while control birds developed severe and more frequent intestinal lesions. The control birds were also showed the clinical signs of reduced feed intake and diarrhea in some cases. However, there were no recorded mortalities during the experiment. No significant statistical difference was observed in gross lesion scores between birds received LCP strain, and NV-challenged birds (*P* = 0.59) in this experiment.

**Table 1:** Gross intestinal lesion scores

Groups	No. of birds	Lesion scores							
		0	1+	2+	3+	4+	5+	6+	Mean
**NV-challenged**	10	-	1	1	5	3	-	-	**3**
**LCP**	10	-	2	1	5	2	-	-	**2.7**
**LC-α**	10	1	5	2	2	-	-	-	**1.5**^**a**^

^a^Immunized group that showed significant reduction in gross intestinal lesion scores compared with control groups, *P* = 0.0058 vs NV-challenged group, *P* = 0.0313 vs LCP group

**Fig. 1:**
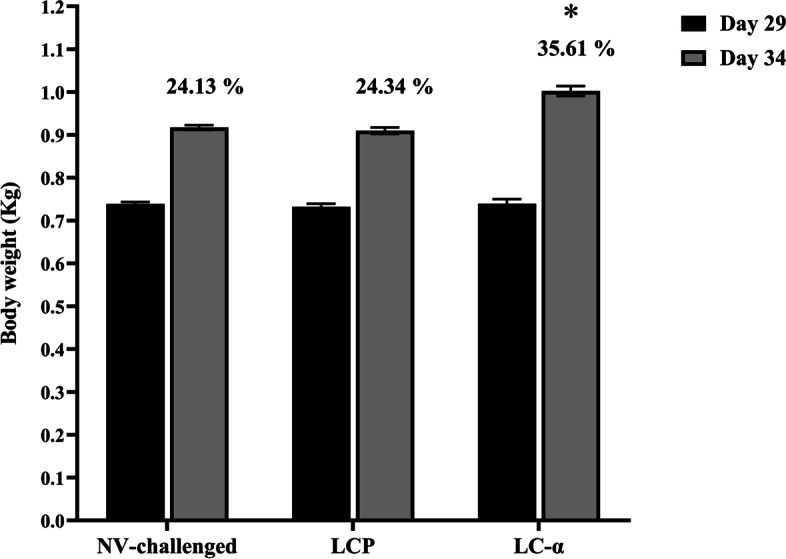
The mean body weights of birds before and after the *C. perfringens* challenge experiment. Birds were individually weighted at 5-day intervals from day 24 to 34. There were no significant statistical differences in mean body weight gains between vaccinated and control birds before the challenge experiment (data not shown). Birds immunized orally with LC-α vaccine strain showed significant body weight gains during the challenge experiment compared with control birds (*P* < 0.0001). Each value indicates mean ± SEM. Asterisk represents significant statistical difference compared with control birds (*P* < 0.0001). All data were analyzed in triplicate

Furthermore, the mean body weight gains measured at 5-day intervals in vaccine and control groups showed no significant statistical differences prior to the challenge experiment (*P* < 0.05). However, birds immunized orally with the LC-α vaccine strain experienced a significant rise in the mean body weight after the challenge experiment compared those received LCP strain or NV-challenged birds (*P* < 0.05) (Fig. 1). There was no significant statistical difference in the mean body weight gains between LCP-received and NV-challenged birds (*P* > 0.999).

### Antibody responses to *C. perfringens* antigen

**Fig. 2:**
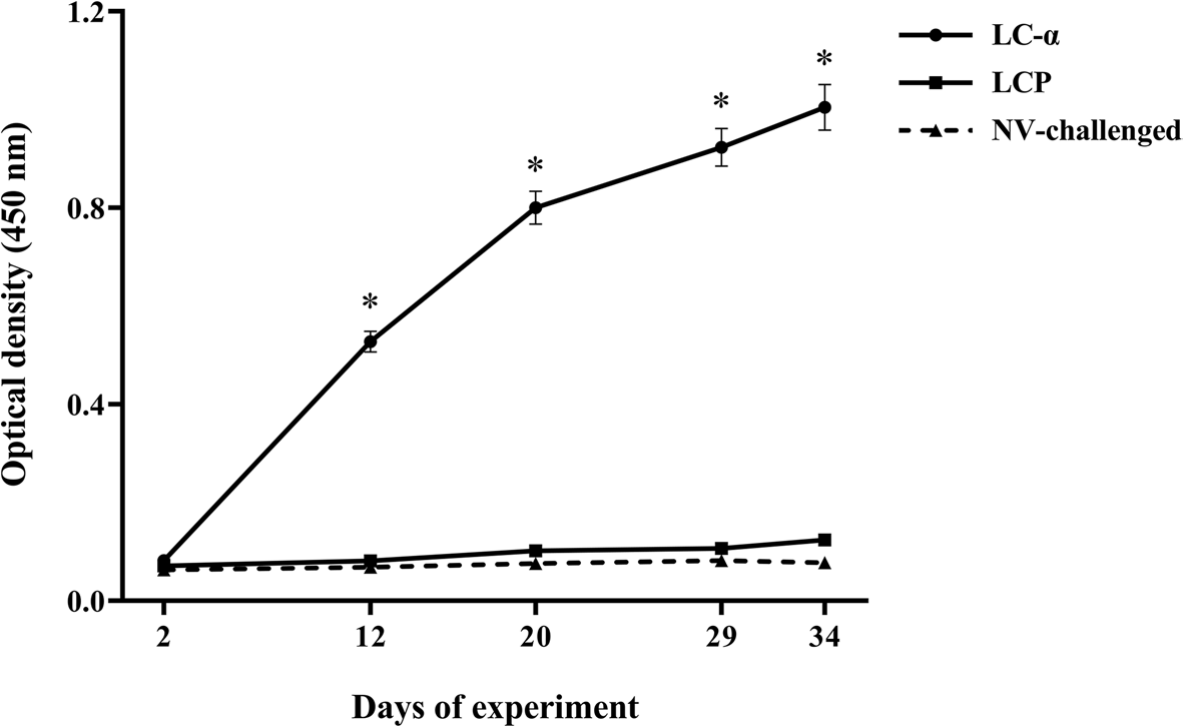
Serum IgY responses of broiler chickens to α-toxin of *C. perfringens*. The standard α-toxin of *C. perfringens* was used as the coating antigen at a density of 25 μg/ml, and the pooled serum anti-α antibodies collected from birds immunized with the recombinant *L. casei* expressing α toxin, birds treated with either *L. casei* harboring empty vector and NV-challenged birds were tested as the source of the primary antibodies at a 1:100 dilution. Goat anti-chicken IgY antibody-HRP was used as the source of the secondary antibody. No significant antibody responses were observed in control birds. * indicates significant values. Values represent mean ± SEM. (*p* < 0.05). All samples were analyzed in triplicate

**Fig. 3:**
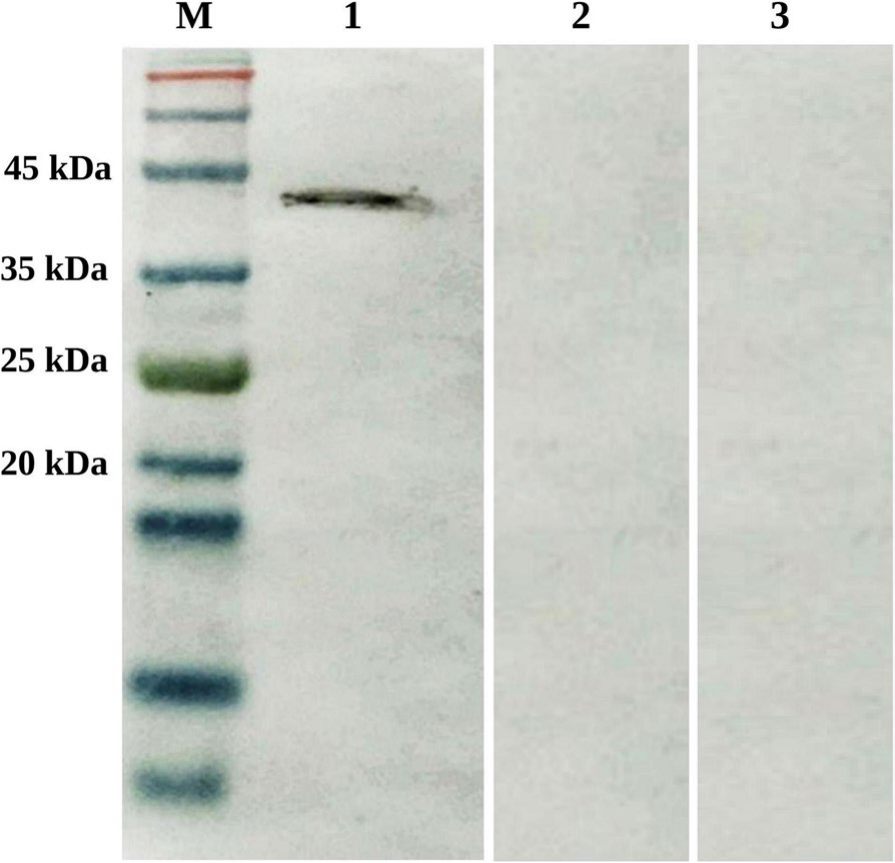
Immunoblot of the standard α-toxin of *C. perfringens* with LC*-*α-immunized chicken serum. Sera collected from chickens immunized with *L. casei* expressing C-terminal domain of α-toxin of *C. perfringens* collected at necropsy were pooled, and then used at a 1:100 dilution. A 43-kDa protein band of the α-toxin of *C. perfringens* (lane 1) shows the reactivity of the chicken anti-α IgY antibodies with α-toxin. No specific reactivity was observed between α-toxin of *C. perfringens* and sera obtained from control birds (lane 2 and 3, birds received LCP strain or NV-challenged birds, respectively). Lane M, the ExcelBand 3-Color Prestained Protein Marker (Smobio, Hsinchu, Taiwan). Full-length blots are presented in Supplementary Fig. 1

The sera collected from birds vaccinated with LC-α vector vaccine were initially tested by an indirect ELISA assay to evaluate the specific anti-α IgY responses after each immunization. Birds immunized orally with recombinant LC-α vaccine strain elicited significant serum antitoxin antibody responses after each vaccination in comparison with pre-immunization level (*P* < 0.05) (Fig. 2). No significant antibody responses were observed in birds received LCP strain as well as birds in the NV-challenged group. Additionally, the presence of the specific anti-α IgY response was also confirmed by immunoblotting. The serum anti-α antibodies obtained from birds immunized orally with LC-α vaccine strain after the last vaccination, showed high affinity with the standard α-toxin immunoblots on the nitrocellulose membrane, and developed the respective protein band of the standard α-toxin with the expected molecular weight (Fig. 3). There was no antibody responses cross reactive to standard α-toxin in the sera collected from birds received LCP strain or NV-challenged birds.

## Discussion

As a result of the withdrawal of growth-promoting antibiotics following the European ban on antibiotics, a number of studies have suggested possible alternative methods for controlling the devastating effects of NE disease on broiler farms and also eliminating antibiotic usage due to public concerns over antibiotic-resistant species arising in poultry-growing countries. Among different alternative approaches to control NE in the post-antibiotic era, vaccination of birds with variety of immunogenic antigens of *C. perfringens* was previously showed to protect birds against experimental challenge of NE [31]. Several studies have reported that parenteral immunization of the broiler chickens with α-toxin of *C. perfringens* could elicit the specific immune responses, and also lower the gross lesions observed in the small intestine of birds after experimental challenge of NE [19, 32–34]. Additionally, it was formerly confirmed that C-terminal domain of α-toxin of *C. perfringens* could be immunogenic and induce antitoxin antibody responses in immunized mice [15, 16] and broiler chickens [18, 19, 35].

The easy route of vaccination is an essential feature that must take into account for developing an appropriate vaccine candidate, particularly for poultry industries with large populations [36]. It is formerly proposed that the administration of live vaccines could induce more robust and permanent immune responses, and also eliminate post-vaccination adverse effects compared with inactive organisms-based vaccines [37, 38]. Some investigators used live oral vector vaccines to immunize birds against experimental challenge of NE. It has been shown that oral inoculation of broiler chickens with an attenuated *salmonella enterica* serovar Typhimurium vaccine strain expressing different immunogenic antigens of *C. perfringens* could elicit significant antitoxin antibody responses in the sera of immunized birds, and also provide partial protection against NE challenge experiment [13, 14, 18, 22].

Probiotics as feed additives were utilized in chickens’ diet and showed the advantages of reducing the severity of lesions observed in the small intestine of birds challenged with NE [23, 24]. These live microorganisms could also prevent pathogens from intestinal colonization and also regulate the mucosal immunity found in the intestine [21, 23]. Furthermore, lactic acid bacteria (LAB) have been widely used as the potential mucosal vaccine vectors against variety of bacterial and viral antigens due to their ability in eliciting strong immune responses [39]. The administration such vaccine constructs could propose many benefits: easy and noninvasive route of administration, high safety levels, cost-effectiveness and capability of high induction of systemic and mucosal immunity against the foreign-expressed antigens [39]. Many investigators, formerly, used LAB, especially *Lactobacillus* spp., as delivery vehicles to express foreign antigens and provide protective systemic immune responses against various avian diseases such as influenza [28, 29, 40, 41], Newcastle disease [30], infectious bursal disease [42] and chicken anemia [27]. In the current study, we used a recombinant *L. casei* carrying a surface-expressed foreign antigen, the C-terminal domain of α-toxin from *C. perfringens*, as an oral probiotic vector vaccine to immunize the broiler chickens and evaluate the potential of this vaccine in providing protection against experimental NE infection. *L. casei* vaccine strain as a safe probiotic bacterium [26] was formerly recognized as a promising vaccine candidate to deliver different foreign immunogens [16, 17]. Being among the safe probiotic microorganisms [26], the *L. casei* vaccine strain has no need for strain attenuation or any required post-vaccination observation for possible adverse effects. Moreover, *L. casei* could also stimulate the immune system, modify the intestinal microbiota, and enhance the growth performance [21, 26].

The broiler chickens vaccinated orally with LC-α vector vaccine and then experimentally infected with virulent *C. perfringens* showed reduction in gross visible lesions in the small intestine. The severity and frequency of the intestinal lesions were reduced in birds immunized with LC-α vaccine strain in comparison with birds received LCP strain or NV-challenged birds. The results also revealed that the birds received vector-only strain or those not-immunized showed mean lesion scores of 2.7 and 3, respectively, while immunization of birds with LC-α vaccine strain (mean lesion score of 1.5) resulted in reduction of the lesion scores to ≤1 in more than half of birds. Additionally, the mean lesion scores observed in birds received LCP strain and also NV-challenged birds showed that the severity of the experimental disease induced in this study was higher in comparison with previous studies which have used the same scoring system and reported scores of 1.07 [43] and 2.6 [44] in control birds. Moreover, birds vaccinated with LC-α vaccine strain had significantly higher mean body weights after the challenge experiment compared with birds in control groups.

In the present study, the ELISA results indicated that the oral immunization of the broiler chickens with LC-α vector vaccine could induce significant anti-α antibody responses compared with control birds, which strengthen the results of the previous studies in relation to the capability of C-terminal domain of α-toxin of *C. perfringens* as an immunogenic antigen.

## Conclusion

In conclusion, the current study showed, for the first time, that a *L. casei* vector vaccine expressing C-terminal fragment of α toxin from *C. perfringens* could be a potential probiotic vector vaccine to provide significant protection against experimental NE challenge in broiler chickens. Our study also indicates and supports the previous findings that the C-terminal fragment of α-toxin of *C. perfringens* is immunogenic, and could be capable of eliciting anti-α antibody responses in broiler chickens and providing protection against experimental NE disease.

## Materials and methods

### Bacterial strains and growth conditions

As the vaccine strain, the LC-α strain, and as the control, the LCP strain, were formerly prepared in our laboratory as described in the previous study [16]. These strains were grown at 37 °C in deMan, Rogosa, and Sharpe (MRS) medium (Himedia, Thane, India) under the anaerobic condition without shaking. Erythromycin (5 μg/ml) was added whenever required. *C. perfringens* strain CP58 was used in the challenge experiment [45]. *C. perfringens* was grown in brain heart infusion (BHI) medium (Merck, Germany) for colony differentiation, and 5% sheep blood agar for hemolytic activity evaluation. Also, cooked meat medium (CMM) (Merck, Darmstadt, Germany), and fluid thioglycolate (FTG) medium (Merck, Darmstadt, Germany) were used to cultivate *C. perfringens* in large quantities, and for animal inoculation in the challenge experiment, respectively. All *C. perfringens* cultures were anaerobically grown at 37 °C without shaking.

### Animals and housing conditions

Commercial day-old Ross 308 broiler chickens were obtained from Mahan Chicken Production Complex (Kerman, Iran) and the parent flock had not received any *C. perfringens* vaccine. The birds were housed in the same room, rearing in similar pens on wood shavings at the density of 20 chicks per 1 m^2^. To avoid contact between different groups, all pens were separated by solid walls. The birds received ad libitum feed and drinking water. In the first week, the chicks were reared at the temperature of 32 °C with a whole-day light program and subsequently, the room temperature decreased 0.5 °C each day to reach 25 °C until the end of the experiment, and the light schedule adjusted to 16 h of light and 8 h of dark for the rest of the period.

### Vaccine preparation

The vaccine and control strains were anaerobically grown in 100 ml of MRS broth at 37 °C after inoculation with 2% v/v inoculum of an overnight culture. Bacterial cells were harvested at an OD_600_ ≥ 2 by centrifugation at 4000×*g* for 10 min at 4 °C. Following washing twice with phosphate-buffered saline (PBS), the pellet was resuspended in PBS until 1 × 10^9^ CFU/ml. The vaccine and control strains were prepared freshly on each day of immunization and were stored on ice until use for inoculation.

### Chicken immunization

**Fig. 4:**
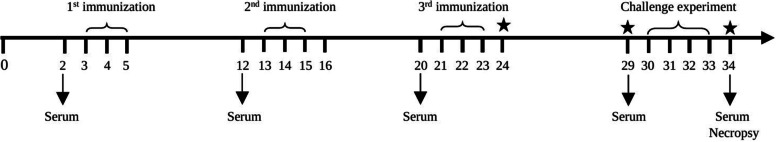
Schedule of vaccination and experimental NE induction in broiler chickens. Birds were immunized orally on days 3, 4, and 5. The second dose of vaccine was given on days 13, 14, and 15, with a boost immunization on days 21, 22, and 23. Serum samples were collected before each immunization, and also 8 and 13 days after the last immunization. Gross examination of the small intestine was carried out 1 day after the challenge experiment. The individual body weights were measured on days 24, 29, and 34 (), at 5-day intervals

The schematic vaccination schedule is shown in Fig. 4. Chickens were randomly allocated to different groups, each comprising 10 birds. On the third day of experiment (4 days of age), birds were orally vaccinated with 0.5 ml of 1 × 10^9^ CFU/ml bacterial suspension of either LC-α or LCP strains for 3 consecutive days. To facilitate the inoculation procedure, all chicks were deprived of feed and water for 12 h prior to each inoculation and 30 min after vaccination the feed and water were returned. Ten days later, birds received 1 ml vaccine or control strains (1 × 10^9^ CFU/bird) for 3 consecutive days. On day 21, all chicks were inoculated with the same volume of LC-α or LCP strains as a boost immunization as before. Additional group of chicks was not vaccinated.

### *C. perfringens* challenge

All birds were fed an antibiotic-free starter diet containing 21.5% protein for 20 days. On day 21, the feed was replaced by a protein-rich feed, a formulated wheat-based grower diet containing 48% fishmeal as the main source of protein.

For experimental infection, *C. perfringens* strain CP58 was grown anaerobically in CMM medium at 37 °C for 24 h. Then, FTG medium was inoculated with 3% of the overnight CMM culture and incubated for 24 h at 37 °C under anaerobic condition. On day 30, chickens that were initially fasted for 12 h, were orally inoculated with 1 ml of 1 × 10^9^ CFU/ml virulent *C. perfringens* culture twice daily (morning and evening). Moreover, the contaminated feed was prepared by mixing an overnight FTG culture with feed at a ratio of 1:2 vol/wt, and was provided to the chickens once per day, immediately after the morning-oral inoculation. The oral and subsequent in-feed challenges were performed for 4 consecutive days. One day after the end of the challenge experiment, all chickens were euthanized by CO2 inhalation and necropsied for postmortem examination.

### Protection assessment

Protection against a 4-day challenge experiment of *C. perfringens* was assessed based on gross lesions in the small intestine at necropsy, and body weight gains of birds before and after the challenge period (Fig. 4). The whole small intestines of all chickens were observed 1 day after the last day of the challenge experiment and the visible lesions were scored using the 1–6 scoring system described previously by Keyburn et al. [46] as follows: 0 = no visible gross lesions; 1 = thin or friable walls; 2 = focal necrosis or ulceration (1–5 foci); 3 = focal necrosis or ulceration (6–15 foci); 4 = focal necrosis or ulceration (16 or more foci); 5 = patches of necrosis (2–3 cm long); 6 = diffuse necrosis similar to that of the field cases. Scorer bias was avoided by using blind scoring. Likewise, the body weights were measured individually from day 24 to 34, at 5-day intervals, for all birds until necropsy.

### Measurement of antibody responses

Blood samples were collected from the left wing veins of all groups during the experiment, as illustrated in Fig. 4, to evaluate the specific antibody responses to *L. casei*-expressed *C. perfringens* antigen in serum samples. An enzyme-linked immunosorbent assay (ELISA) was performed to determine the presence of antigen-specific immunoglobulin Y (IgY) in immunized birds. The polystyrene 96-well microtiter plates (Nunc, Roskilde, Denmark) were coated with 25 μg/ml of phospholipase C™ (α-toxin) from *C. perfringens* (Sigma, St. Louis, US) diluted in coating solution (PBS, pH 7.4), and then incubated overnight at 4 °C. Blocking step was done at 37 °C for 2 h with PBS containing 3% skimmed milk (Sigma, St. Louis, USA) to eliminate the nonspecific binding. As the source of the primary antibodies, pooled chicken sera were diluted in PBS (1:100), added in duplicate and then incubated for 1 h at 37 °C. Goat anti-chicken IgY antibody-HRP (Genscript, Piscataway, USA) (diluted 1:3000 in PBS-3% skimmed milk) was added as the secondary antibodies followed by incubation for 1 h at 37 °C. The color reaction was developed using TMB substrate. After incubation for 15 min at room temperature, the reaction was stopped using 1 M H_2_SO_4_ and the absorbance at 450 nm was measured using an ELISA plate reader (Biotek, Vermont, USA).

The serum anti-toxin antibody responses in birds immunized with LC-α strain were also confirmed using western blot analysis. Firstly, the standard α-toxin (10 μg) was run on a 12% SDS-polyacrylamide gel and then transferred onto a nitrocellulose membrane. After the blocking step, the blots were reacted with pooled serum antibodies (1:100 in PBS) collected from birds vaccinated with the LC-α vaccine strain. The reaction of α-toxin with pooled antibodies from birds received LCP strain and also NV-challenged birds was examined to confirm that control groups did not develop any antibodies cross reactive to standard α-toxin. Goat anti-chicken IgY antibody-HRP (1:3000 diluted in PBS) was used as the source of the secondary antibodies, and the color reaction was carried out using 4-chloro-1-naphthol (Sigma, St. Louis, USA) substrate.

### Statistical analysis

All statistics were carried out using GraphPad Prism 9.0 (Graph-Pad Software, San Diego, CA). Antibody titers and body weight values were analyzed using two-way analysis of variance followed by Tukey’s posttest. Lesion scores were analyzed using a two-tailed Mann-Whitney test. Values were expressed as means ± SEM, and p < 0.05 was considered the significance level. All data were analyzed in triplicate.

## Supplementary Information

**Additional file 1: Additional figure 1.** Uncropped full-length blots. Lane M, the ExcelBand 3-Color Prestained Protein Marker; lane 1, the 43-kDa protein band of the α-toxin of *C. perfringens*; lane 2, birds received LCP strain; lane 3, NV-challenged birds. Unrelated lanes are indicated with ×.

**Additional file 2.** ELISA data of vaccinated and control birds. The ELISA data of the sera collected from birds on days 2, 12, 20, 29, and 34 are shown in 450 nm optical density.

**Additional file 3. **Challenge data of vaccinated and control birds. The lesion scores of birds challenged with virulent *C. perfringens* are shown using the 1–6 scoring system*.*

**Additional file 4.** Individual body weights of chickens (kg). Body weights of birds measured on days 24, 29, and 34 are shown.

## Data Availability

All data generated or analyzed during this study are included in this published article [and its supplementary information files].
