# Supplementary figures and images for: Evaluation the efficacy of oral immunization of broiler chickens with a recombinant Lactobacillus casei vaccine vector expressing the Carboxy-terminal fragment of α-toxin from Clostridium perfringens
Source: BMC Vet Res. 2023 Jan 19;19:13. doi: 10.1186/s12917-023-03566-8 (PMC9850811; doi:10.1186/s12917-023-03566-8)

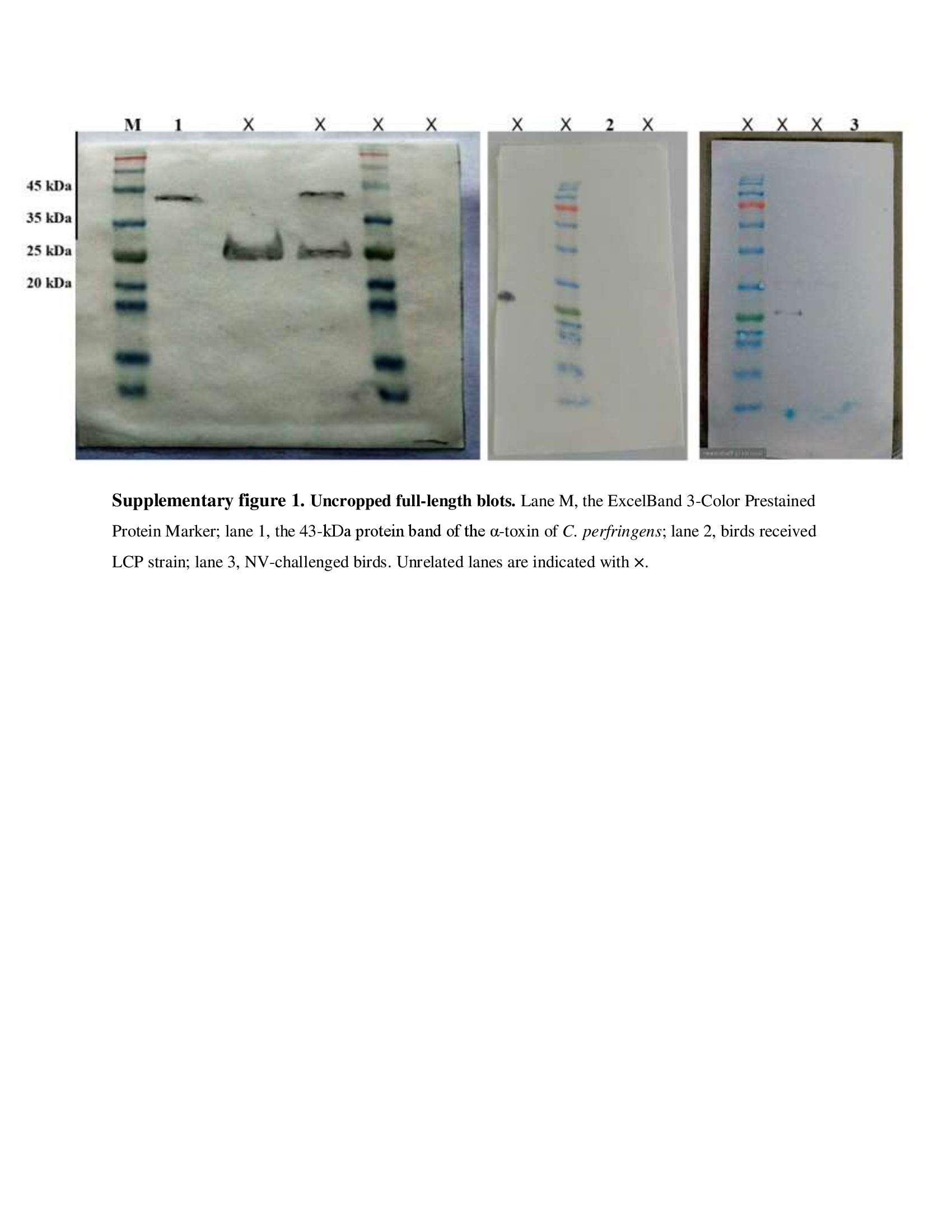

Supplement: Supplementary file 1 — Additional file 1: Additional figure 1. Uncropped full-length blots. Lane M, the ExcelBand 3-Color Prestained Protein Marker; lane 1, the 43-kDa protein band of the α-toxin of C. perfringens; lane 2, birds received LCP strain; lane 3, NV-challenged birds. Unrelated lanes are indicated with ×. [file 12917_2023_3566_MOESM1_ESM.tiff]
